# Supplementary material for: An open-source framework for large-scale, flexible evaluation of biomedical text mining systems
Source: J Biomed Discov Collab. 2008 Jan 29;3:1. doi: 10.1186/1747-5333-3-1 (PMC2276192; doi:10.1186/1747-5333-3-1)
Supplement: Additional file 1 — Evaluation platform source code. The additional file contains the software evaluation framework discussed in this paper. The most current release can be downloaded from SourceForge.net [1]. See the accompanying README file for instructions on installation and use. Also included in the distribution is a collection of UIMA wrappers for some commonly used BioNLP tools and annotated corpora. [file 1747-5333-3-1-S1.zip › bionlp_uima_repository-1.0.1/docs/api/edu/uchsc/ccp/uima/cc/summary/comparison/AnnotationGroup.html]

AnnotationGroup (Center for Computational Pharmacology Util/UIMA API)


|  |  |  |
| --- | --- | --- |
| **Overview** \| **Package** \| **Class** \| **Tree** \| **Deprecated** \| **Index** \| **Help** | |  |
| **PREV CLASS**   **NEXT CLASS** | **FRAMES**    **NO FRAMES**     **All Classes** |  |
| SUMMARY: NESTED \| FIELD \| CONSTR \| METHOD | DETAIL: FIELD \| CONSTR \| METHOD |  |


---


## edu.uchsc.ccp.uima.cc.summary.comparison Class AnnotationGroup

```
java.lang.Object
  edu.uchsc.ccp.uima.cc.summary.comparison.AnnotationGroup
```

---

``` public class AnnotationGroup extends Object ```

---

**Constructor Summary**

`AnnotationGroup()`


| **Method Summary** | |
| --- | --- |
| `void` | `addAnnotationType(String type)` |
| `Integer` | `getAnnotationSetID()` |
| `List<String>` | `getAnnotationTypeList()` |
| `Integer` | `getAnnotatorID()` |
| `AnnotationGroupID` | `getGroupID()` |
| `boolean` | `isValid()` |
| `void` | `print(PrintStream ps)` |
| `void` | `setAnnotationSetID(Integer annotationSetID)` |
| `void` | `setAnnotationTypeList(List<String> annotationTypeList)` |
| `void` | `setAnnotatorID(Integer annotatorID)` |
| `void` | `setGroupID(AnnotationGroupID groupID)` |

**Methods inherited from class java.lang.Object**

`clone, equals, finalize, getClass, hashCode, notify, notifyAll, toString, wait, wait, wait`

**Constructor Detail**

### AnnotationGroup

```
public AnnotationGroup()
```


**Method Detail**

### isValid

```
public boolean isValid()
```

---


### getAnnotationSetID

```
public Integer getAnnotationSetID()
```

---


### setAnnotationSetID

```
public void setAnnotationSetID(Integer annotationSetID)
```

---


### getAnnotationTypeList

```
public List<String> getAnnotationTypeList()
```

---


### setAnnotationTypeList

```
public void setAnnotationTypeList(List<String> annotationTypeList)
```

---


### addAnnotationType

```
public void addAnnotationType(String type)
```

---


### getAnnotatorID

```
public Integer getAnnotatorID()
```

---


### setAnnotatorID

```
public void setAnnotatorID(Integer annotatorID)
```

---


### getGroupID

```
public AnnotationGroupID getGroupID()
```

---


### setGroupID

```
public void setGroupID(AnnotationGroupID groupID)
```

---


### print

```
public void print(PrintStream ps)
```


---


|  |  |  |
| --- | --- | --- |
| **Overview** \| **Package** \| **Class** \| **Tree** \| **Deprecated** \| **Index** \| **Help** | |  |
| **PREV CLASS**   **NEXT CLASS** | **FRAMES**    **NO FRAMES**     **All Classes** |  |
| SUMMARY: NESTED \| FIELD \| CONSTR \| METHOD | DETAIL: FIELD \| CONSTR \| METHOD |  |


---
